# Exploring perceptions of healthcare technologies enabled by artificial intelligence: an online, scenario-based survey

**DOI:** 10.1186/s12911-021-01586-8

**Published:** 2021-07-20

**Authors:** Alison L. Antes, Sara Burrous, Bryan A. Sisk, Matthew J. Schuelke, Jason D. Keune, James M. DuBois

**Affiliations:** 1 Bioethics Research Center, Washington University School of Medicine in St. Louis, St. Louis, MO USA; 2 Department of Pediatrics, Division of Hematology and Oncology, Washington University School of Medicine in St. Louis, St. Louis, MO USA; 3 Division of Biostatistics, Washington University School of Medicine in St. Louis, St. Louis, MO USA; 4 Departments of Surgery and Health Care Ethics, Bander Center for Medical Business Ethics, Saint Louis University, St. Louis, MO USA

**Keywords:** Artificial intelligence, Machine learning, Acceptance of healthcare, Openness, Benefits, Concerns, Perceptions, Bioethics

## Abstract

**Background:**

Healthcare is expected to increasingly integrate technologies enabled by artificial intelligence (AI) into patient care. Understanding perceptions of these tools is essential to successful development and adoption. This exploratory study gauged participants’ level of openness, concern, and perceived benefit associated with AI-driven healthcare technologies. We also explored socio-demographic, health-related, and psychosocial correlates of these perceptions.

**Methods:**

We developed a measure depicting six AI-driven technologies that either diagnose, predict, or suggest treatment. We administered the measure via an online survey to adults (N = 936) in the United States using MTurk, a crowdsourcing platform. Participants indicated their level of openness to using the AI technology in the healthcare scenario. Items reflecting potential concerns and benefits associated with each technology accompanied the scenarios. Participants rated the extent that the statements of concerns and benefits influenced their perception of favorability toward the technology. Participants completed measures of socio-demographics, health variables, and psychosocial variables such as trust in the healthcare system and trust in technology. Exploratory and confirmatory factor analyses of the concern and benefit items identified two factors representing overall level of concern and perceived benefit. Descriptive analyses examined levels of openness, concern, and perceived benefit. Correlational analyses explored associations of socio-demographic, health, and psychosocial variables with openness, concern, and benefit scores while multivariable regression models examined these relationships concurrently.

**Results:**

Participants were moderately open to AI-driven healthcare technologies (M = 3.1/5.0 ± 0.9), but there was variation depending on the type of application, and the statements of concerns and benefits swayed views. Trust in the healthcare system and trust in technology were the strongest, most consistent correlates of openness, concern, and perceived benefit. Most other socio-demographic, health-related, and psychosocial variables were less strongly, or not, associated, but multivariable models indicated some personality characteristics (e.g., conscientiousness and agreeableness) and socio-demographics (e.g., full-time employment, age, sex, and race) were modestly related to perceptions.

**Conclusions:**

Participants’ openness appears tenuous, suggesting early promotion strategies and experiences with novel AI technologies may strongly influence views, especially if implementation of AI technologies increases or undermines trust. The exploratory nature of these findings warrants additional research.

**Supplementary Information:**

The online version contains supplementary material available at 10.1186/s12911-021-01586-8.

## Introduction

Recent advances in machine learning have prompted widespread enthusiasm about the potential for artificial intelligence (AI) to transform healthcare [1–6]. As Rajkomar, Dean, and Kohane assert:

…the wisdom contained in the decisions made by nearly all clinicians and the outcomes of billions of patients should inform the care of each patient. …machine learning is not just a new tool…it is the fundamental technology required to meaningfully process data that exceed the capacity of the human brain to comprehend…. [2] p1347

Accompanying this enthusiasm towards AI are concerns about realizing these promises, while recognizing unintended perils [7–15]. As Israni and Verghese noted, “The promise of AI is undeniable…the hype and fear surrounding the subject are greater than that which accompanied the discovery of the structure of DNA or the whole genome.” [7] p29.

AI health technologies are already influencing healthcare practices. Applications developed for screening skin cancer, oral cancer, and Tuberculosis offer hope that more people will be able to access screening tools that could dramatically alter care [16–18]. The FDA has approved a number of AI-enabled devices, including devices that detect wrist fractures and diabetic retinopathy [19, 20]. The adoption of these technologies must consider the perspectives of patients, as their effective implementation requires them to engage with AI technologies and share their health data [21–24]. Available, albeit limited, research examining patient perspectives of AI technologies in healthcare suggests that patients perceive both benefits and risks and have mixed willingness to adopt these technologies.

A study in France examined views of biometric monitoring devices (BMDs) and their integration into healthcare among 1183 patients with chronic conditions [25]. They estimated that just 20% of patients viewed the possible benefits, such as access to care and reduced treatment burden, as greatly outweighing the potential risks, such as AI being a poor alternative to humans and mishandling of private data. Participants indicated their readiness to use four BMD and AI technologies in their own care. The majority (65%) was ready to incorporate all of the interventions, but, for many, only if humans managed their use. Few (3%) were ready for fully automated use. Another 22% of patients were against one of the technologies, and 13% were not ready for any of the technologies.

A study from PricewaterhouseCoopers of 12,000 people from Europe, the Middle East, and Africa, found that 54% of participants were willing to engage with AI and robotic technologies, 38% were not willing to engage with these technologies, and 7% were indifferent [26]. The findings also revealed that the purpose of the technologies influenced participants’ willingness to use them. While 37% of participants were willing to have AI or a robot monitor a heart condition and advise on treatment, only 1% were willing to have these technologies deliver a baby. However, a survey of patient satisfaction with a specific application of AI for diabetic retinopathy screening in Australia found that 96% of patients were satisfied or very satisfied with automated screenings, with convenience being particularly important to them [27].

In semi-structured interviews with dermatology patients in the United States (U.S.), the most common perceived advantages of AI were increased diagnostic speed, healthcare access, and diagnostic accuracy [28]. However, patients also viewed the possibility of less accurate diagnosis to be the greatest potential disadvantage, and 94% of participants preferred human-AI partnership to AI alone. Another qualitative study of patients in the Netherlands regarding AI in radiology concluded that patients’ knowledge is limited and education may be required to foster acceptance of AI and obtain patient input on implementation [29]. A preliminary framework of patients’ perspectives produced by the study indicated patients were concerned with efficiency, accountability, reliability, and the boundaries of technologies relative to human providers.

These prior studies did not assess openness or perceived concerns and benefits regarding AI technologies in healthcare among individuals in the U.S., or examine potential correlates that might help understand perceptions. The context and characteristics of the healthcare tasks enabled by AI may influence perceptions [30]. Individuals may have different views of AI that enables diagnosis, treatment, or prognosis, and these views may especially depend on the seriousness, consequences, and complexity of the decision-making required [31]. For example, individuals may respond differently to applications that diagnose or treat cancer versus a broken bone. Individuals may also hold different views related to AI-enabled technologies they might utilize at home—e.g., personalized health Apps and wearables—whereas they might prefer to engage with humans in the clinic or hospital. Further, whether the tools aim to promote wellness or provide treatment—e.g., reduce risk of heart disease versus treat heart disease—may influence perceptions.

In addition to perceptions potentially depending, at least in part, on task context and characteristics, individuals may perceive certain risks or benefits associated with AI-enabled technologies in healthcare. For example, the potential for AI to improve the efficiency and accuracy of decisions may be appealing, but the potential loss of professional discretion and individualized interactions may be concerning [30, 32]. Applications of AI in society generally have raised concerns about their potential to undermine fairness and further exacerbate inequities [31]. A recent report from the National Academy of Medicine indicates that equity and inclusion must be prioritized when designing and scaling AI, as consumer-facing technologies in other domains have exacerbated longstanding inequities [33]. Thus, individuals might perceive concerns related to social justice with the advent of AI-enabled tools in healthcare.

## Objective

The biomedical community will need to understand individuals’ perceptions of AI-enabled health technologies as they are developed and adopted into patient care. The purpose of this study was to develop a novel measure and assess openness and the extent of perceived concerns and benefits regarding AI-driven healthcare technologies in a sample of U.S. adults. In additional to assessing levels of openness, concern, and benefit, we explored associations with socio-demographic, health, and psychosocial variables to identify variables that might help explain these perceptions. We conducted this exploratory study using a crowdsourcing platform, Amazon Mechanical Turk (MTurk). MTurk offers access to a geographically dispersed set of respondents who can be more representative and diverse than locally collected samples [34], but respondents tend to be relatively young, digitally savvy adults [35, 36]. Thus, the current study reflects views that are not necessarily representative of the broader U.S. population. We viewed MTurk as a feasible and acceptable platform for this initial, exploratory study [37], and are careful to interpret our findings in light of the exploratory nature of the study.

## Methods

### Preliminary work to develop new measure

We developed the “Perceptions of AI Technologies in Healthcare” measure to assess openness and perceived concerns and benefits. We chose to design a scenario-based measure so that participants’ perceptions would be contextualized in light of realistic examples of AI applications in healthcare. The measure development team consisted of physicians, social scientists with expertise in bioethics and psychometrics, and a healthcare social worker. The term “artificial intelligence” was not included in the measure (or the study more broadly) to avoid misconceptions or preconceived ideas about AI. Instead, we used terms such as technology and computer programs because we wanted to study perceptions of the functionality and potential uses of these applications rather than views of the concept of artificial intelligence. The measure development process included informant interviews, a literature review, drafting and revising items, and factor analysis of items after a first round of data collection.

We included a range of AI-enabled healthcare applications. Scenarios varied in the emotional intensity (e.g., broken ankle vs cancer), purpose of the AI-driven technology (i.e., diagnosis, treatment, or prognosis), and setting for use of the device (i.e., hospital, doctor’s office, and at home). The initial measure included eight scenarios, and the refined version included six scenarios. Table S1 shows all six scenarios (see Additional file 1). An example scenario includes:

Your doctor has diagnosed you with colon cancer. The cancer clinic has a computer program that uses the medical information of thousands of patients with colon cancer to estimate survival. This computer reviews your medical information and predicts you have a very low chance of surviving more than six months.

After each scenario, participants indicated their level of openness to the described use of technology on a 5-point Likert scale from: *not at all open* (1) to *extremely open* (5). We defined openness as being receptive to the use of the technology in one’s care. Next, each scenario included concern and benefit items describing 1 of 9 ethical and practical concerns or benefits associated with AI in healthcare.

We identified the concerns and benefits through informant interviews and a literature review. We interviewed 7 experts working on AI in the fields of bioinformatics, law, bioethics, and medicine. We asked how they define AI, how they describe AI to laypersons, to provide examples of current and possible future uses of AI in healthcare, and to list concerns and benefits likely to be salient to patients. We created an initial set of concerns and benefits gleaned by identifying themes in the interviews. Then, we referenced these concerns and benefits against issues raised in a literature review of applications of AI in healthcare and associated ethical, social, and legal issues.

To conduct this review, we consulted a medical librarian and created search strategies for PubMed, Scopus, and Embase to obtain articles about “artificial intelligence,” “machine learning,” “big data,” and “healthcare.” After this broader search, we conducted two narrower searches by adding search terms related to ethics and patient perspectives. The literature review resulted in more than 300 articles about the practical and ethical aspects of AI in healthcare, including review articles and commentaries underscoring key ethical issues [12–15, 38–41]. The interviews and literature review resulted in 9 dimensions—5 concerns and 4 benefits—that individuals may perceive regarding AI in healthcare. Table 1 shows the dimensions and their definitions, which we used to operationalize the dimensions in the measure.

**Table 1:** Dimensions operationalized in the scenario-based measure

	Definition
Concerns	
Privacy	Concern about loss of control of personal information, misuse of information, and who can access personal information [14, 25, 38, 42–45]
Transparency/uncertainty	Concern about the comprehensibility of AI results or recommendations and uncertainty about being made aware when AI is used in healthcare [12, 14, 38, 42]
Human element of care	Concern about AI decreasing the clinician’s role in healthcare and these technologies impacting the interactions and relationships of clinicians and patients [9, 25, 46–48]
Social justice	Concern about unfairness in the distribution of the benefits and burdens of applications of AI in healthcare [6, 12–14, 38, 49–52]
Cost for healthcare system	Concern about whether AI applications will increase the costs of healthcare delivery in the U.S. [11, 53]
Benefits	
Access and convenience	Perceived benefit of AI making it easier for individuals to obtain medical care [16–18, 54, 55]
Quality and accuracy	Perceived benefit of AI applications increasing the effectiveness of medical care [56–59]
Access to personal health knowledge	Perceived benefit of easily obtaining reliable and pertinent information outside of the clinical setting for use to improve personal health [26, 38]
Improving personal cost of care	Perceived benefit that AI could reduce the costs of healthcare for individuals [60–62]

When a topic could be a potential concern or benefit (e.g., AI could improve or impair accuracy), we included it as a concern or benefit according to which the literature review and interviews suggested would be most salient to individuals. We wrote items for all of these dimensions to adequately cover the full range of possible concerns and benefits. However, we expected that factor analysis of responses would likely factor items into a set of fewer dimensions.

The measure operationalized the concerns and benefits in items that followed each scenario. Example items include, “Your insurance company charges you an additional copay to use this program” (personal cost) and “Using the computer program makes your visit to urgent care shorter” (convenience). Participants responded on a bi-polar, 7-point Likert scale from *much more negatively* (1) to *much more positively* (7) to indicate the extent each statement influenced their perception of the technology. The initial version of the measure had 54 concern and benefit items with at least 5 items for each concern and benefit to allow us to discard any poorly performing items identified in the factor analysis.

After drafting the measure, two bioinformaticians reviewed it to provide feedback on the technical accuracy and plausibility of the AI scenarios. We also performed cognitive interviews with 5 members of the community who were diverse in age, race/ethnicity, and education level to receive feedback on item clarity [63]. Table S2 provides a scenario with the associated items to illustrate the structure of the scenarios, items, and response scales (see Additional file 2).

### Design and procedure

We administered the Perceptions of AI Technologies in Healthcare measure and several additional validated measures (described in “Variables and measures”) online using Qualtrics survey administration platform. We recruited participants via MTurk who were individuals 18 years of age and older and residing in the U.S. MTurk is a platform that connects individuals who complete “human intelligence tasks” (HITs) with requestors. We indicated our task was a survey of views of health technologies and compensation was $3.65 for the 30-min task. Requestors determine the amount compensation for their HIT, which tends to be below minimum wage. We paid minimum wage for the 30-min task. MTurk has been shown to produce valid results comparable to those from laboratory studies [35, 64]. We required participants to have completed at least 100 prior HITs with a 98% approval rating for their completion of previous tasks.

We collected data in two rounds to perform an exploratory factor analysis (EFA) of the concern and benefit items, followed by a confirmatory factor analysis to verify the results of the EFA. Assuming we identified as many as 6 factors and retained at least 5 items per factor, even assuming low levels of communality, a sample size of 400 for a factor analysis allows for excellent agreement between the sample and population solutions [65, 66]. Thus, we sought a sample size of at least 400 for the EFA, and chose to obtain an equally large sample for the CFA to confirm the solution and provide a large sample for exploration of variables associated with responses.

The Washington University Institutional Review Board reviewed and approved this study (IRB #201909088). Consent was obtained from all participants. Participants viewed a brief consent statement on the first screen of the survey before proceeding, which indicated their consent to participate. Data were collected in October of 2019.

### Variables and measures

In this section, we describe the measures included and our rationale. Each respondent provided a full set of response data at a single point in time. Although common source bias is a concern when measurement is conducted using a single instrument [67], we were interested in individuals’ perceptions, and the most direct way to measure perceptions is through survey methodology. We mitigated common source bias via careful survey design [67, 68]; we measured the openness, concern, and benefit variables on different scales with different anchors using a scenario-based measurement task, whereas trust and personality variables were measured using traditional validated psychosocial questionnaires.

#### Perceptions of AI Technologies in Healthcare

The key outcome variables of interest included openness to AI in healthcare, and perceived concerns and benefits of AI in healthcare. We measured these variables using the new scenario-based measure described above. We randomized the presentation order of the scenarios and the concern and benefit items within scenarios to control for potential order effects. The results describe the factor analysis of concern and benefit items, and the internal consistency of the openness, concern, and benefit scales. After refining the measure based on the factor analysis, we retained 22 concern items and 16 benefit items. We computed an overall concern score as the mean of the 22 concern items, after reverse scoring so that higher scores reflect a greater level of concern. We computed an overall benefit score as the mean of the 16 benefits items, with greater scores on this scale reflecting greater levels of perceived benefit. Overall, concern and benefit scores can range from 1 to 7. We computed the mean of the 6 openness items to produce an overall openness score, which can range from 1 to 5.

#### Ten Item Personality Inventory (TIPI)

The TIPI measures five personality traits: openness to experience, conscientiousness, extraversion, agreeableness, and emotional stability [64]. Participants responded on a 7-point scale from 1 “strongly disagree” to 7 “strongly agree” to indicate whether ten pairs of traits (e.g., reserved, quiet) apply to them. The five scales are computed as the mean of the two items for each. We included this brief measure to examine the association of openness to AI technologies assessed by our new measure with trait-based openness. We also aimed to explore if other personality traits might be associated with perceptions of novel technologies in healthcare, as other studies have identified relationships between personality and health behaviors. For example, conscientiousness has been associated with health promoting behaviors [69].

#### Trust in health information systems

The trust in health systems and health information sharing measure includes items related to four sub-scales: fidelity, competency, trust, and integrity [70]. An example item includes, “The organizations that have my health information and share it would try to hide a serious mistake.” A 4-point Likert scale is used: 1 “not at all true” to 4 “very true.” The four sub-scales are computed as the mean of items for that subscale. We computed the composite “health system trust index” score for use in analyses, which is the sum of the four subscales (each with a possible range of 1 to 4), so potential scores on the index can range from 4 to 16 [70]. We included this measure of trust in the healthcare system expecting that trust might be associated with greater openness to healthcare innovations and greater perceived benefit, and negatively associated with concerns.

#### Trust and faith in general technology

A brief faith in general technology and trust in technology scale was included [71]. Example items include: “I think most technologies enable me to do what I need to do” and “I usually trust a technology until it gives me a reason not to trust it.” Participants use a 7-point Likert scale to respond from 1 “strongly disagree” to 7 “strongly disagree.” An overall score for each scale was the mean of the respective items in the scale. We anticipated a positive association of trustful attitudes towards technology with perceived benefits and openness, and a negative association with concerns.

#### Social and economic conservatism scale

We included a conservatism scale that measures both social and economic conservatism [72]. Participants responded on a sliding 0 to 100 point scale (in 10-point increments), with 0 representing a negative view and 100 indicating a positive view of 12 concepts (e.g., business, traditional values). Social and economic conservatism scores were computed as the mean of the concepts representing each construct. We included this scale to explore if social conservatism and economic conservatism might be associated lower openness and greater concerns about changes in healthcare.

#### Health status and healthcare access

We assessed self-reported health status, healthcare satisfaction, primary insurance type, location of health services, and amount of healthcare choice using existing items [73]. The response options for these 1-item categorical variables are displayed in the Table 1 frequencies. We thought that experiences with healthcare might relate to perceptions about new healthcare technologies.

#### Socio-demographics

We included a questionnaire assessing age, sex, employment status, income, ethnicity, race, education level, and the type of community where participants reside.

### Statistical analysis

Data cleaning involved examining responses to four “attention check” items included in the Perceptions of AI Technologies in Healthcare measure to identify participants who did not pay sufficient attention. We required that participants answer at least three of four attention checks correctly. Before analyses were performed, individuals failing two or more attention checks were excluded. In round one, 50 responses were dropped and 46 were dropped in round two. Force choice responding was used for the AI measure so we had no missing data on this measure.

The sample from round one of data collection was used to perform an exploratory factor analysis (EFA) to examine the factor structure of the 54 concern and benefit items and identify items that did not perform well. The sample from round two was used to perform a confirmatory factor analysis (CFA) to confirm the initial factor solution. We also examined scale internal consistency, and we report Cronbach’s alpha for the retained concern and benefit items and for the openness items. We used descriptive statistics to summarize participant characteristics. Because we failed to detect any statistically significant differences between the two samples on perceptions of AI technologies or the socio-demographic, health, and psychosocial variables, the remaining analyses focused on the aggregated sample.

We used descriptive statistics to examine openness to the AI technologies illustrated in the six scenarios responded to by all participants. We also used descriptive statistics to assess overall levels of openness, concern, and perceived benefit. Next, we used correlations to explore bivariate associations of socio-demographic, health, and psychosocial variables to levels of openness, concern, and perceived benefit. Finally, we performed three stepwise linear regressions with the openness, concern, and benefit variables as the outcomes. This analysis allowed us to explore the variables as potential predictors in the context of the other variables. We entered age, sex, race, and ethnicity as control variables in a first step of each model. Next, all other socio-demographic, health status and access, and psychosocial variables were included for consideration as predictors using stepwise R^2^ criteria for predictor variable entry and removal (probability-of-F-to-enter ≤ 0.05; probability-of-F-to-remove ≥ 0.10). Healthcare satisfaction was the one variable excluded from consideration because we only asked it of individuals who had utilized healthcare in the last 12 months (n = 735), and its inclusion would have reduced the effective sample size considerably.

## Results

### Description of participants

A total of 936 individuals participated. Table 2 summarizes their socio-demographic and healthcare characteristics. Participants were mostly White, healthy, college-educated individuals. On average, participants were in their mid-thirties.

**Table 2:** Participant socio-demographics and healthcare variables

	Sample 1 (N = 469)	Sample 2 (N = 467)	Total (N = 936)
Age in years	M = 37.2 ± 11.0 Range 65, 18–83	M = 36.9 ± 11.0 Range 53, 19–72	M = 37.1 ± 11.0 Range 65, 18–83

	n (%)	n (%)	n (%)
Sex (male)^a^	256 (55)	258 (55)	514 (55)
Race/ethnicity^b,c^			
White	383 (82)	398 (85)	781 (83)
Black or African American	52 (11)	44 (9)	96 (10)
Latino or Hispanic	37 (8)	36 (8)	73 (8)
Asian	36 (8)	30 (6)	66 (7)
Other^d^	7 (2)	10 (2)	17 (2)
Highest education			
Less than high school or other	6 (1)	2 (< 1)	8 (1)
High school graduate	57 (12)	63 (14)	120 (13)
Some college	100 (21)	112 (24)	212 (23)
Associate’s degree	48 (10)	63 (14)	111 (12)
Bachelor’s degree	205 (44)	181 (39)	386 (41)
Graduate degree	53 (11)	46 (10)	99 (11)
Employment status			
Employed full-time	329 (70)	308 (66)	637 (68)
Employed part-time (not full-time student)	28 (6)	30 (6)	58 (6)
Full-time student	11 (2)	11 (2)	22 (2)
Self-employed	47 (10)	64 (14)	111 (12)
Unemployed	22 (5)	23 (5)	45 (5)
Other^e^	32 (7)	31 (7)	63 (7)
Annual household income^f^			
< $23,000	75 (16)	57 (12)	132 (14)
$23,001–$45,000	118 (25)	148 (32)	266 (28)
$45,001–$75,000	139 (30)	134 (29)	273 (29)
$75,001–$112,000	87 (19)	81 (17)	168 (18)
> $112,001	46 (10)	40 (9)	86 (9)
Type of community			
Urban	139 (30)	126 (27)	265 (28)
Suburban	242 (52)	242 (52)	484 (52)
Rural	88 (19)	99 (21)	187 (20)
Health status			
Excellent	83 (18)	69 (15)	152 (16)
Very good	154 (33)	174 (37)	328 (35)
Good	155 (33)	139 (30)	294 (31)
Fair	57 (12)	70 (15)	127 (14)
Poor	20 (4)	15 (3)	35 (4)
Primary health insurance type			
Private	294 (63)	258 (55)	552 (59)
Medicare	39 (8)	48 (10)	87 (9)
Medicaid	52 (11)	62 (13)	114 (12)
Medicare advantage	11 (2)	14 (3)	25 (3)
No health insurance	73 (16)	85 (18)	158 (17)
Typical healthcare service location			
Doctor’s office or private clinic	324 (69)	291 (62)	615 (66)
Urgent care center	59 (13)	74 (16)	133 (14)
Community health center or other public health clinic	25 (5)	37 (8)	62 (7)
No regular place of care	32 (7)	48 (10)	80 (9)
Hospital emergency room	18 (4)	8 (2)	26 (3)
Other	11 (2)	9 (2)	20 (2)
Medical care choice^g^			
A great deal of choice	123 (26)	99 (21)	222 (24)
Some choice	236 (50)	237 (51)	473 (51)
Very little choice	83 (18)	100 (21)	183 (20)
No choice	22 (5)	19 (4)	41 (4)
Healthcare satisfaction^h^			
Very satisfied	157 (34)	132 (28)	289 (31)
Somewhat satisfied	171 (37)	196 (42)	367 (39)
Somewhat dissatisfied	38 (8)	26 (6)	64 (7)
Very dissatisfied	7 (2)	8 (2)	15 (2)

Some percentages add to more than 100%, due to rounding

^a^n = 11 selected other or prefer not to answer

^b^not mutually exclusive categories, participants selected all that apply

^c^n = 8 selected prefer not to answer

^d^American Indian, Alaska Native, Native Hawaiian, or Pacific Islander

^e^caregiver or homemaker, retired, or other

^f^n = 11 selected prefer not to answer

^g^n = 17 selected “I don’t know”

^h^only asked of those indicating healthcare utilization in last 12 months (n = 735)

### Factor analyses of AI concern and benefit items

The Appendix provides the full description of the EFA and CFA results (see Additional file 3). In sample one, we found a factor solution with two orthogonal factors. Factor 1 had 22 items and represented participants’ level of concern and accounted for 22% of the variance. Factor 2 had 16 items and represented participants’ level of perceived benefit and accounted for 18% of the variance. This model reflects dropping 16 items that either did not load on the factors, or were from two scenarios that we dropped at this stage in their entirety. We made this decision to decrease the participant burden because the additional items were redundant for a simple 2-factor solution. Table S3 provides the factor loadings of the final 38-item solution (see Additional file 4). The CFA in sample 2 confirmed this factor structure with acceptable model fit.

### Descriptive analyses of perceptions of AI technologies in healthcare

**Fig. 1:**
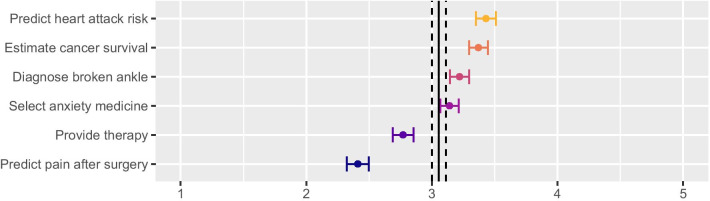
Mean scenario openness scores with 95% CIs. Large vertical lines indicate grand mean with 95% CI

Figure 1 illustrates the openness scores by scenario. Participants were most open to the scenario about monitoring for heart attack risk (M = 3.40, SD = 1.20) followed by predicting cancer survival (M = 3.37, SD = 1.16), diagnosing a broken ankle (M = 3.22, SD = 1.20), and selecting anxiety medication (M = 3.14, SD = 1.16). We observed the lowest openness for the mental health app (M = 2.77, SD = 1.29) and a computer system that uses video to monitor facial expressions and predict pain levels in a hospital room after surgery (M = 2.41, SD = 1.35). All pairwise comparisons (with alpha adjustment for multiple comparisons) are statistically significant (p < 0.01). The SDs for all openness scores were above 1, suggesting considerable variability in openness.

Table 3 shows the descriptive statistics and Cronbach’s alpha for the overall level of openness, concern, and perceived benefit scores, along with the psychosocial variables. The mean concern score indicates that concern statements led participants to report that they viewed the technology somewhat more negatively. When participants rated benefit items, they similarly reported somewhat more positive views of the technology. On average, participants reported moderate openness to the technologies.

**Table 3:** Descriptives for openness, concern, and benefit scores and psychosocial variables

	No. of items	Cronbach’s α	Min	Max	Mean	SD	95% CI for mean
Openness^a^	6	.80	1.0	5.0	3.06	.87	[3.00, 3.12]
Concern	22	.92	1.2	7.0	5.34	.82	[5.29, 5.39]
Benefit	16	.89	2.6	7.0	5.49	.75	[5.44, 5.54]
Health System Trust Index	20	.91	4.0	16.0	9.48	2.63	[9.31, 9.65]
Trust in technology	3	.89	1.0	7.0	4.95	1.32	[4.87, 5.03]
Faith in technology	4	.87	1.0	7.0	5.56	.83	[5.51, 5.61]
Conscientiousness	2	.67	1.5	7.0	5.59	1.23	[5.51, 5.67]
Agreeableness	2	.55	1.0	7.0	5.37	1.30	[5.29, 5.45]
Extraversion	2	.80	1.0	7.0	3.37	1.77	[3.26, 3.48]
Emotional stability	2	.82	1.0	7.0	4.91	1.64	[4.80, 5.02]
Openness (trait-based)	2	.61	1.0	7.0	5.08	1.34	[4.99, 5.17]
Social conservatism	7	.90	0.0	100.0	55.77	25.65	[54.13, 57.40]
Economic conservatism	5	.73	0.0	100.0	53.63	20.53	[52.31, 54.95]

N = 936, except for agreeableness (n = 935), emotional stability (n = 934), faith in technology (n = 934), trust in technology (n = 933) due to missing data

^a^Correlations between the Perspective of AI Technologies scores: Openness with concern, r = − .52, 95% CI [− .57, − .47]; openness with benefit, r = .61, 95% CI [.57, .65]; concern with benefit, r = − .05 CI [− .11, − .01]

### Correlational analyses

The correlational analyses shown in Table 4 explored which variables were associated with openness, concern, and benefit scores. Correlations are Point-biserial, Spearman, or Pearson depending on the variable measurement scale. We focused our interpretation of correlations on those ≥ 0.10.

**Table 4:** Correlations of openness, concern, and benefit scores with all study variables

	Openness		Concern		Benefit	
	r	95% CI	r	95% CI	r	95% CI
*Socio-demographics*						
Age	− .12	[− .18, − .06]	.06	[.00, .12]	− .03	[− .09, .03]
Sex (1 = Male, 0 = Female)	.10	[.04, .16]	− .20	[− .26, − .14]	− .03	[− .09, .04]
Race (1 = White, 0 = Non-White)^a^	− .05	[− .11, .01]	.01	[− .05, .07]	− .08	[− .14, − .02]
Ethnicity (1 = Latino, 0 = non-Latino)	.06	[.00, .12]	− .09	[− .15, − .03]	− .02	[− .08, .04]
Household income	.07	[.01, .13]	− .08	[− .14, − .02]	.07	[.01, .13]
Community type	.06	[.00, .12]	− .06	[− .12, .00]	.01	[− .05, .07]
Employment status^b^	.17	[.11, .23]	− .18	[− .24, − .12]	.05	[− .01, .11]
Education	.04	[− .02, .10]	.03	[− .03, .09]	.01	[− .05, .07]
*Health status and access*						
Health status	.08	[.02, .14]	− .12	[− .18, − .06]	− .02	[− .08, .04]
Healthcare location^c^	.03	[− .03, .09]	− .01	[− .07, .05]	.02	[− .04, .08]
Healthcare choice^d^	.08	[.02, .14]	− .06	[− .12, .00]	.11	[.05, 17]
Health insurance (1 = Yes, 0 = No)	.09	[.03, .15]	− .10	[− .16, − .04]	.05	[− .01, .11]
Healthcare satisfaction (n = 735)	.11	[.04, .18]	− .07	[− .14, .00]	.14	[.07, .21]
*Psychosocial variables*						
Health System Trust Index	.27	[.21, .33]	− .27	[− .33, − .21]	.21	[.15, .27]
Trust in technology	.41	[.36, 46]	− .21	[− .27, − .15]	.41	[.36, .46]
Faith in technology	.38	[.32, .43]	− .10	[− .16, − .04]	.46	[.41, .51]
Conscientiousness	.02	[− .04, .08]	.11	[.05, .17]	.15	[.09, .21]
Agreeableness	.08	[.02, .14]	.11	[.05, .17]	.20	[.14, .26]
Extraversion	.08	[.02, .14]	− .12	[− .18, − .06]	.04	[− .02, .10]
Emotional stability	.08	[.02, .14]	− .06	[− .12, .00]	.07	[.01, .13]
Openness (trait-based)	.07	[.01, .13]	.07	[.01, .13]	.05	[− .01, .11]
Social conservatism	− .01	[− .07, .05]	− .10	[− .16, − .04]	.05	[− .01, .11]
Economic conservatism	− .06	[− .12, .00]	− .06	[− .12, .00]	.02	[− .04, .08]

N = 936 (except as noted for specific variables in Tables 1 and 2)

^a^Participants who selected any race other than White, or in addition to White, were classified as Non-White for purposes of this analysis

^b^1 = full-time employment, 0 = all other options

^c^1 = doctor office or private clinic, 0 = all other options

^d^1 = great or some choice; 0 = little to no choice

This exploratory analysis of variables associated with openness, concern, and perceived benefit indicated that socio-demographic and health variables were largely unrelated. There were modest relationships of age and sex to openness: older participants were less open and males more open than females. Females also responded more negatively when presented with concerns. Full-time employment status was associated with greater openness and lower concern. People with greater healthcare choice and healthcare satisfaction perceived greater benefit, and lower health status was associated with greater concern.

The openness score was minimally associated with the trait-based personality measure of openness (*r* = 0.07), suggesting responses did not merely capture participants’ general tendency towards openness. Personality generally was not strongly related to perceptions of AI in healthcare. Agreeableness and conscientiousness were the strongest correlates with those higher in agreeableness and conscientiousness perceiving greater benefit. Social conservatism was related to lower concern scores but only slightly. Trust in health and trust and faith in technology were the strongest correlates of openness, concern, and benefit scores, with these correlations being about 1.5 to over 4.0 times the magnitude of the other variables that were associated at *r* ≥ 0.10.

### Regression analyses

As shown in Table 5, the regression models revealed that some of the same predictors of openness, concern, and benefit were important across all three outcomes, while other predictors were statistically significant in just one or two of the models. In each model, certain social-demographic and health-related variables were statistically significant predictors, but similar to the correlational analyses, we observed the largest effects for psychosocial predictors.

**Table 5:** Stepwise regression models predicting openness, concern, and benefit

Predictor	Model 1: Openness			Model 2: Concern			Model 3: Benefit		
	B	95% CI	β	B	95% CI	β	B	95% CI	β
Age	− 0.01	[− 0.01, 0.00]	− 0.07*	0.00	[0.00, 0.00]	0.00	0.00	[− 0.01, 0.00]	− 0.03
Sex	0.08	[− 0.02, 0.18]	0.05	− 0.22	[− 0.32, − 0.12]	− 0.13***	− 0.07	[− 0.15, 0.02]	− 0.04
Race	− 0.01	[− 0.14, 0.11]	− 0.01	− 0.00	[− 0.12, 0.12]	− 0.00	− 0.12	[− 0.22, − 0.01]	− 0.06*
Ethnicity	0.05	[− 0.14, 0.23]	0.01	− 0.15	[− 0.33, 0.03]	− 0.05	− 0.11	[− 0.26, 0.05]	− 0.04
Employment status	0.27	[0.16, 0.37]	0.14***	− 0.24	[− 0.35, − 0.13]	− 0.14***			
Health status				− 0.23	[− 0.36, − 0.09]	− 0.11**			
Health system trust	0.04	[0.02, 0.06]	0.12***	− 0.06	[− 0.08, − 0.04]	− 0.20***			
Trust in technology	0.17	[0.12, 0.21]	0.25***	− 0.10	[− 0.14, − 0.06]	− 0.16***	0.12	[0.08, 0.16]	0.22***
Faith in technology	0.22	[0.15, 0.29]	0.21***				0.30	[0.24, 0.37]	0.34***
Conscientiousness	− 0.06	[− 0.10, − 0.02]	− 0.09**	0.12	[0.08, 0.16]	0.18***			
Agreeableness				0.10	[0.06, 0.14]	0.15***			
Extraversion				− 0.04	[− 0.07, − 0.01]	− 0.08**			
Social conservatism				0.00	[0.00, 0.00]	− 0.07*			
Economic conservatism	0.00	[− 0.01, 0.00]	− 0.07*						
R^2^	.26***			.21***			.25***		

N = 916. Age, sex, race, and ethnicity entered in a first step as control variables. Age is continuous. Variables are coded as follows: Sex (1 male; 0 female), race (1 White; 0 non-White), and ethnicity (1 Latino; 0 not-Latino). Employment status (1 full-time; 0 other); health status (1 good/very good/excellent; 0 poor/fair)

*p < .05; **p < .01; ***p < .001

In the model predicting openness, trust in technology and faith in technology were associated with greater openness. Next, full-time employment and trust in the health system were moderately associated with greater openness, while being older, more conscientious, and more economically conservative were modestly associated with less openness. The overall model explained 26% of the variance in openness.

In the model predicting concern, we found that health system trust and trust in technology were associated with lower concern, while conscientiousness and agreeableness were associated with greater concern. Males were also less concerned than females. Employment status and health status were negatively related to concern. Finally, we found modest associations of extraversion and social conservatism, such that individuals higher in extraversion and social conservatism were less concerned. The overall model explained 21% of the variance in concern.

The model predicting benefit indicated that greater trust and faith in technology predicted greater perceived benefit. There was a modest association with race, with White individuals perceiving lower benefit than non-White. The overall variance explained for the model predicting benefit was 25%.

## Discussion

We examined perceptions of AI-driven healthcare technologies using a scenario-based measure of openness, concern, and perceived benefit. We assessed overall openness across six varied applications of AI in healthcare. Within each scenario, concern items related to loss of privacy, lack of transparency, decreased clinician role in care, increased costs, and unfairness in the benefits for different groups (e.g., female versus male, or White patients versus people of color), whereas benefit items focused on access and convenience, increased quality of care, improved healthcare costs, and access to personal health knowledge. We also measured a number of socio-demographic, health-related, and psychosocial variables to understand what might explain openness, concern, and perceived benefit. We collected data using MTurk, a crowdsourcing platform, which provides feasible, cost-effective access to geographically dispersed individuals, but our findings should be interpreted in the context of our sample.

We constructed a sample composed entirely of U.S. residents, which may limit the generalizability of our findings in other countries, because we wished to examine perceptions of individuals sharing a common national health system. Our sample proved to be comprised of relatively young, healthy, White adults, which does not represent all subpopulations in the U.S. However, in our large sample of over 900 individuals, the sufficient variance in age, self-reported health status, and race allowed us to identify some associations of these factors with perceptions of AI-enabled healthcare technologies, and these findings persisted even after controlling for variables like trust in healthcare. Older individuals were less open than younger individuals; males were less concerned than females; and full-time employment status was associated with greater openness and lower concern. Individuals reporting good to excellent health were less concerned, so examining perceptions among those with lower health status will be important. The findings suggest further examination of which socio-demographic and health-related variables influence acceptance of AI technologies is warranted.

Overall, participants were moderately open to the technologies, with some variation in opinion based on the specific application. The two technologies that made predictions about serious diseases—the risk of heart attack and the likelihood of cancer survival—were the highest-rated technologies. Openness to these uses of AI may be partly due to familiarity. These are high prevalence diseases, and the majority of Americans report frequent exposure to information about prevention of these diseases [74]. Participants were least open to a device that predicted pain after surgery and a mental health app. Lower openness to these uses of AI could relate to perceptions of invasiveness, desire for human involvement, or stigma related to pain medication and mental health treatment.

Trust in the healthcare system and trust and faith in technology had the strongest, most consistent relationships to openness to AI healthcare technologies and to judgments of potential benefits and harms. Plans for the development and implementation of AI in healthcare will need to consider ways to build and maintain trust. It may also be important to examine how interpersonal trust with individual physicians may shape behaviors and attitudes related to AI technologies [75, 76]. The association of trust with perceptions of AI in healthcare is notable as in recent years Americans report decreased trust in the healthcare system and lower confidence in physicians [77].

Some personality variables emerged as predictors of perceptions. In particular, conscientiousness and agreeableness demonstrated effects similar to those of trust in predicting concern. Individuals high in conscientiousness tend to be responsible and goal-directed, and conscientiousness is related to better health and greater well-being [69]. The concern items, especially those depicting loss of privacy and lack of transparency, may have been particularly troubling to those high in conscientiousness. Agreeableness is associated with interpersonal warmth, understanding, and compassion [78], so the social justice items illustrating unfairness and the items depicting loss of interpersonal interaction with healthcare providers might account for greater concern. If personality traits are involved in perceptions and acceptance of new AI-enabled healthcare technologies, this fact might be somewhat challenging to address given personality tends to be fixed in adulthood. Likewise, conservatism reflects a relatively stable set of deeper political and social beliefs, and while only weakly related to perceptions in this study, the potential for these beliefs to influence perceptions is worthy of further consideration.

It is also worth noting the typical response pattern when we presented participants with potential concerns and perceived benefits of AI technologies in healthcare and asked them to report how much these issues swayed their perceptions. Overall, participants reported a slight downtick towards more negative views when presented with concerns, and a slight uptick in favorability when presented with benefits. The benefits elicited a slightly stronger increase in perceptions than the decrease produced by concerns, which may suggest the importance of highlighting benefits of these technologies. However, the increase relative to the decrease in perceptions caused by concerns was small, and thus may not be clinically significant. It will be necessary in future research to disentangle the relative risks and benefits that participants perceive and which tradeoffs, if any, they are comfortable with and in which healthcare contexts. A qualitative approach allowing participants to respond to healthcare scenarios in an open-ended fashion might be fruitful.

Moreover, we wrote items representing different types of concerns and benefits aiming to identify those that created the most worry and greatest enthusiasm. We anticipated participants would respond to distinct types of benefits and concerns (e.g., quality, privacy, and cost), and our cognitive interviews indicated participants distinguished the different domains addressed by the questions. However, factor analysis revealed two underlying response patterns reflecting a general extent of concern and perceived benefit. It appears that participants responded to the benefit/concern (i.e., positive/negative) framing of the items, not necessarily to an evaluation of the specific *underlying* concern or benefit.

It could be that the positive/negative framing highlighted the emotional salience of the statements, so a general affective response (e.g., “I like or do not like that”) guided responses. Participants were also fairly young and likely to be digitally savvy [36]. They may be familiar with similar benefits (e.g., convenience and quality) and concerns (e.g., cost and privacy) in other technologies generally, thus the various benefits and concerns may not elicit different response patterns. On the other hand, this pattern of responding in general versus with attention to the specific issues might indicate that perceptions of these technologies are relatively tenuous, perhaps due to limited knowledge or experience with such technologies in healthcare.

The way these technologies are promoted to the public is likely to be highly significant in fostering openness and positive perceptions. Early experiences patients have with AI-driven healthcare technologies will also likely have a strongly influence on views. When presented with novel, unfamiliar technologies, patients will need to trust the recommendations arising from these tools and engage with information provided by physicians [79]. In some cases, patients will need to directly engage with new tools, often in a sustained fashion over time [80]. To maximize the potential of these AI tools in healthcare, it is important to involve users and patient perspectives. Interdisciplinary collaborations among technology developers, informaticians, social scientists, and clinicians, and patient engagement experts will be best suited for this task in both the development and adoption stages [7, 81]. Implementation strategies can also be used to improve adoption, implementation, and sustainability of novel technologies in clinical care [82]. It will also be essential to address underrepresentation of certain populations in data and in uptake of new health technologies to address the potential for such tools to exacerbate long-standing health disparities [22, 33, 50].

## Limitations

Again, these findings should be considered in light of study limitations. In this exploratory study, we focused on obtaining a U.S. sample via MTurk. This approach offers a sample often more diverse than other sources of data but not truly representative of the U.S. population [34]. MTurk allowed us to obtain a cost effective, large sample of adults who live across the U.S., but follow-up studies should explore perspectives among samples that reflect greater diversity in race/ethnicity, community type (i.e., urban–rural), and educational levels. We also recommend further consideration of the potential for less favorable perceptions among older individuals, women, and those without full-time employment.

It is also of note that the cross-sectional nature of the study does not indicate if these views are stable over time. Additionally, MTurk participants may be particularly at ease with technologies and potentially more open. Our method yielded scores reflecting overall extent of concern and perceived benefit, though we aimed to elucidate views towards different kinds of concerns and benefits. Patients might demonstrate different views of distinct concerns and benefits if perceptions were measured in a different manner. For instance, if participants were asked to prioritize which of the concerns and benefits they viewed as most important relative to others.

Finally, it is difficult to completely rule out and address the potential for common source bias. For example, there is the potential for positive affectivity bias to jointly influence trust and openness in the measurement of perceptual variables [67, 83]. As described in the methods, we addressed common source bias through survey design, measuring the outcome variables using different scales and tasks than the predictor variables [68]. It is also notable that the variables examined here accounted for 21–26% of the variance in the outcomes of interest, suggesting that additional variables will need to be identified to fully understand perceptions of AI-enabled healthcare technologies.

## Conclusion

Although the study has some limitations, the research provides a novel scenario-based approach to examining views that might be adapted in future studies. We found that a sample of relatively young U.S. adults was moderately open to the AI-driven technologies presented in the healthcare scenarios. We further identified that it may be essential to attend to trust when aiming to foster acceptance of these novel healthcare innovations. Finally, we provided evidence that a combination of socio-demographics, health-related, and psychosocial variables may contribute to individuals’ perceptions and hope this study stimulates additional research.

## Supplementary Information

**Additional file 1: Table S1.** Scenarios from the perceptions of AI technologies in healthcare measure.

**Additional file 1: Table S2.** Example scenario with openness, concern, and benefit items.

**Additional file 3.** Full results of the exploratory and confirmatory factor analysis.

**Additional file 4: Table S3.** Results of the final 38-item exploratory factor analysis conducted in sample 1 (N = 469).

## Data Availability

The datasets used and analyzed during the current study are available from the corresponding author on reasonable request.

## Abbreviations

AI: Artificial intelligence
EFA: Exploratory factor analysis
CFA: Confirmatory factor analysis

## References

[1] Jiang F, Jiang Y, Zhi H, Dong Y, Li H, Ma S (2017). Artificial intelligence in healthcare: past, present and future. Stroke Vasc Neurol.

[2] Rajkomar A, Dean J, Kohane I (2019). Machine learning in medicine. N Engl J Med.

[3] Burgess M (2018). Now deepmind's ai can spot eye disease just as well as your doctor.

[4] Dolins SB, Kero RE, editors. The role of ai in building a culture of partnership between patients and providers. AAAI Spring Symposium—Technical Report; 2006.

[5] Li D, Kulasegaram K, Hodges BD (2019). Why we needn't fear the machines: opportunities for medicine in a machine learning world. Acad Med.

[6] Topol EJ (2019). High-performance medicine: the convergence of human and artificial intelligence. Nat Med.

[7] Israni ST, Verghese A (2019). Humanizing artificial intelligence. JAMA.

[8] Mukherjee S (2017). A.I. versus m.D.

[9] Becker A (2019). Artificial intelligence in medicine: what is it doing for us today?. Health Policy Technol.

[10] JASON (2017). Artificial intelligence for health and health care.

[11] Maddox TM, Rumsfeld JS, Payne PRO (2019). Questions for artificial intelligence in health care. JAMA.

[12] Reddy S, Allan S, Coghlan S, Cooper P (2019). A governance model for the application of ai in health care. J Am Med Inform Assoc.

[13] Char DS, Shah NH, Magnus D (2018). Implementing machine learning in health care—addressing ethical challenges. N Engl J Med.

[14] Vayena E, Blasimme A, Cohen IG (2018). Machine learning in medicine: addressing ethical challenges. PLoS Med.

[15] McDougall RJ (2019). Computer knows best? The need for value-flexibility in medical ai. J Med Ethics.

[16] Esteva A, Kuprel B, Novoa RA, Ko J, Swetter SM, Blau HM (2017). Dermatologist-level classification of skin cancer with deep neural networks. Nature.

[17] Lopez-Garnier S, Sheen P, Zimic M (2019). Automatic diagnostics of tuberculosis using convolutional neural networks analysis of mods digital images. PLoS ONE.

[18] Uthoff RD, Song B, Sunny S, Patrick S, Suresh A, Kolur T (2018). Point-of-care, smartphone-based, dual-modality, dual-view, oral cancer screening device with neural network classification for low-resource communities. PLoS ONE.

[19] Fda permits marketing of artificial intelligence-based device to detect certain diabetes-related eye problems [press release]. April 11, 2018; 2018.

[20] Fda permits marketing on artifical intelligence algorithm for aiding providers in detecting wrist fractures [press release]. 2018.

[21] Shaw J, Rudzicz F, Jamieson T, Goldfarb A (2019). Artificial intelligence and the implementation challenge. J Med Internet Res.

[22] McCradden MD, Joshi S, Anderson JA, Mazwi M, Goldenberg A, Zlotnik SR (2020). Patient safety and quality improvement: Ethical principles for a regulatory approach to bias in healthcare machine learning. J Am Med Inform Assoc.

[23] Lennon MR, Bouamrane MM, Devlin AM, O'Connor S, O'Donnell C, Chetty U (2017). Readiness for delivering digital health at scale: lessons from a longitudinal qualitative evaluation of a national digital health innovation program in the United Kingdom. J Med Internet Res.

[24] Wagner JK, Peltz-Rauchman C, Rahm AK, Johnson CC (2016). Precision engagement: the pmi's success will depend on more than genomes and big data. Genet Med.

[25] Tran V-T, Riveros C, Ravaud P (2019). Patients’ views of wearable devices and ai in healthcare: findings from the compare e-cohort. NPJ Digit Med.

[26] PricewaterhouseCoopers. What doctor? Why ai and robotics will define new health. 2017.

[27] Keel S, Lee PY, Scheetz J, Li Z, Kotowicz MA, MacIsaac RJ (2018). Feasibility and patient acceptability of a novel artificial intelligence-based screening model for diabetic retinopathy at endocrinology outpatient services: a pilot study. Sci Rep.

[28] Nelson CA, Pérez-Chada LM, Creadore A, Li SJ, Lo K, Manjaly P (2020). Patient perspectives on the use of artificial intelligence for skin cancer screening: a qualitative study. JAMA Dermatol.

[29] Haan M, Ongena YP, Hommes S, Kwee TC, Yakar D (2019). A qualitative study to understand patient perspective on the use of artificial intelligence in radiology. J Am Coll Radiol.

[30] Bullock JB (2019). Artificial intelligence, discretion, and bureaucracy. Am Rev Public Adm.

[31] Young MM, Bullock JB, Lecy JD (2019). Artificial discretion as a tool of governance: a framework for understanding the impact of artificial intelligence on public administration. Perspect Public Manag Governance.

[32] Busch PA, Henriksen HZ (2018). Digital discretion: a systematic literature review of ict and street-level discretion. Inf Polity.

[33] Matheny M, Israni ST, Ahmed M, Whicher D (2019). Artificial intelligence in health care: the hope, the hype, the promise, the peril.

[34] Huff C, Tingley D (2015). "Who are these people?" Evaluating the demographic characteristics and political preferences of mturk survey respondents. Res Polit.

[35] Mason W, Suri S (2012). Conducting behavioral research on amazon's mechanical turk. Behav Res Methods.

[36] Munger K, Luca M, Nagler J, Tucker J. Everyone on mechanical turk is above a threshold of digital literacy: Sampling strategies for studying digital media effects. Working Paper. https://csdp.princeton.edu/sites/csdp/files/media/munger…; 2018.

[37] Stritch JM, Pedersen MJ, Taggart G (2017). The opportunities and limitations of using mechanical turk (mturk) in public administration and management scholarship. Int Public Manag J.

[38] Fenech M, Strukelj N, Buston O (2018). Ethical, social, and political challenges of artificial intelligence in health.

[39] Luxton DD (2014). Recommendations for the ethical use and design of artificial intelligent care providers. Artif Intell Med.

[40] Yu KH, Beam AL, Kohane IS (2018). Artificial intelligence in healthcare. Nat Biomed Eng.

[41] Yu KH, Kohane IS (2019). Framing the challenges of artificial intelligence in medicine. BMJ Qual Saf.

[42] Balthazar P, Harri P, Prater A, Safdar NM (2018). Protecting your patients' interests in the era of big data, artificial intelligence, and predictive analytics. J Am Coll Radiol.

[43] Price WN (2018). Big data and black-box medical algorithms. Sci Transl Med.

[44] Price WN, Cohen IG (2019). Privacy in the age of medical big data. Nat Med.

[45] Price WN (2017). Artificial intelligence in health care: applications and legal implications. SciTech Lawyer.

[46] Banks J (2018). The human touch: Practical and ethical implications of putting ai and robotics to work for patients. IEEE Pulse.

[47] Mittelman M, Markham S, Taylor M (2018). Patient commentary: stop hyping artificial intelligence - patients will always need human doctors. BMJ (Online).

[48] Verghese A, Shah NH, Harrington RA (2018). What this computer needs is a physician: humanism and artificial intelligence. JAMA.

[49] Ferryman K, Winn RA. Artificial intelligence can entrench disparities-here's what we must do. The Cancer Letter. 2018. https://cancerletter.com/articles/20181116_1/.

[50] Gianfrancesco MA, Tamang S, Yazdany J, Schmajuk G (2018). Potential biases in machine learning algorithms using electronic health record data. JAMA Intern Med.

[51] Nordling L (2019). A fairer way forward for ai in health care. Nature.

[52] Adamson AS, Smith A (2018). Machine learning and health care disparities in dermatology. JAMA Dermatol.

[53] Emanuel EJ, Wachter RM (2019). Artificial intelligence in health care: will the value match the hype?. JAMA.

[54] Meskó B, Hetényi G, Gyorffy Z (2018). Will artificial intelligence solve the human resource crisis in healthcare?. BMC Health Serv Res.

[55] Tsay D, Patterson C (2018). From machine learning to artificial intelligence applications in cardiac care. Circulation.

[56] Fujisawa Y, Otomo Y, Ogata Y, Nakamura Y, Fujita R, Ishitsuka Y (2019). Deep-learning-based, computer-aided classifier developed with a small dataset of clinical images surpasses board-certified dermatologists in skin tumour diagnosis. Br J Dermatol.

[57] Haenssle HA, Fink C, Schneiderbauer R, Toberer F, Buhl T, Blum A (2018). Man against machine: diagnostic performance of a deep learning convolutional neural network for dermoscopic melanoma recognition in comparison to 58 dermatologists. Ann Oncol.

[58] Raumviboonsuk P, Krause J, Chotcomwongse P, Sayres R, Raman R, Widner K (2019). Deep learning versus human graders for classifying diabetic retinopathy severity in a nationwide screening program. NPJ Digit Med.

[59] Urban G, Tripathi P, Alkayali T, Mittal M, Jalali F, Karnes W (2018). Deep learning localizes and identifies polyps in real time with 96% accuracy in screening colonoscopy. Gastroenterology.

[60] Golding LP, Nicola GN (2019). A business case for artificial intelligence tools: the currency of improved quality and reduced cost. J Am Coll Radiol.

[61] Mori Y, Kudo S, East JE, Rastogi A, Bretthauer M, Misawa M (2020). Cost savings in colonoscopy with artificial intelligence—aided polyp diagnosis: an add-on analysis of a clinical trial (with video). Gastrointest Endosc.

[62] Liew C (2018). The future of radiology augmented with artificial intelligence: a strategy for success. Eur J Radiol.

[63] Peterson CH, Peterson NA, Powell KG (2017). Cognitive interviewing for item development: validity evidence based on content and response processes. Meas Eval Couns Dev.

[64] Buhrmester M, Kwang T, Gosling SD (2011). Amazon's mechanical turk: a new source of inexpensive, yet high-quality, data?. Perspect Psychol Sci.

[65] Mundfrom DJ, Shaw DG (2005). Minimum sample size recommendations for conducting factor analyses. Int J Test.

[66] MacCallum RC, Widaman KF, Zhang S, Hong S (1999). Sample size in factor analysis. Psychol Methods.

[67] Favero N, Bullock JB (2015). How (not) to solve the problem: an evaluation of scholarly responses to common source bias. J Public Adm Res Theory.

[68] Podsakoff PM, MacKenzie SB, Podsakoff NP (2012). Sources of method bias in social science research and recommendations on how to control it. Annu Rev Psychol.

[69] Atherton OE, Robins RW, Rentfrow PJ, Lamb ME (2014). Personality correlates of risky health outcomes: findings from a large internet study. J Res Pers.

[70] Platt JE, Jacobson PD, Kardia SLR (2018). Public trust in health information sharing: a measure of system trust. Health Serv Res.

[71] McKnight DH, Choudhury V, Kacmar C (2002). Developing and validating trust measures for e-commerce: an integrative typology. Inf Syst Res.

[72] Everett JAC (2013). The 12 item social and economic conservatism scale (secs). PLoS ONE.

[73] Commonwealth Fund. Health care quality survey 2002. https://www.commonwealthfund.org/publications/surveys/2002/mar/2001-health-care-quality-survey.

[74] Funk C, Kennedy B, Hefferon M (2017). Vast majority of americans say benefits of childhood vaccines outweigh risks.

[75] Iott BE, Campos-Castillo C, Anthony DL. Trust and privacy: how patient trust in providers is related to privacy behaviors and attitudes. In: AMIA Annual Symposium proceedings AMIA Symposium. 2020;2019. p. 487–93. PMC7153104 32308842

[76] Sisk B, Baker JN. A model of interpersonal trust, credibility, and relationship maintenance. Pediatrics. 2019. 10.1542/peds.2019-1319 PMC6889969 31722962

[77] Blendon RJ, Benson JM, Hero JO (2014). Public trust in physicians—U.S. Medicine in international perspective. N Engl J Med.

[78] DeYoung CG, Weisberg YJ, Quilty LC, Peterson JB (2013). Unifying the aspects of the big five, the interpersonal circumplex, and trait affiliation. J Pers.

[79] Diprose WK, Buist N, Hua N, Thurier Q, Shand G, Robinson R (2020). Physician understanding, explainability, and trust in a hypothetical machine learning risk calculator. J Am Med Inform Assoc.

[80] Milne-Ives M, van Velthoven MH, Meinert E (2020). Mobile apps for real-world evidence in health care. J Am Med Inform Assoc.

[81] Petersen C, Austin RR, Backonja U, Campos H, Chung AE, Hekler EB (2019). Citizen science to further precision medicine: from vision to implementation. JAMIA Open.

[82] Proctor EK, Powell BJ, McMillen JC (2013). Implementation strategies: recommendations for specifying and reporting. Implement Sci.

[83] George B, Pandey SK (2017). We know the yin—but where is the yang? Toward a balanced approach on common source bias in public administration scholarship. Rev Public Person Adm.

