# Supplementary material for: Exploring perceptions of healthcare technologies enabled by artificial intelligence: an online, scenario-based survey
Source: BMC Med Inform Decis Mak. 2021 Jul 20;21:221. doi: 10.1186/s12911-021-01586-8 (PMC8293482; doi:10.1186/s12911-021-01586-8)
Supplement: Supplementary file 1 — Additional file 1: Table S1. Scenarios from the perceptions of AI technologies in healthcare measure. [file 12911_2021_1586_MOESM1_ESM.docx]

| **Table S1.**  Scenarios from the Perceptions of AI Technologies in Healthcare Measure |
| --- |
| Diagnose broken ankle  You twisted your ankle and go to an urgent care center. A doctor orders an X-ray. A computer program reviews your X-ray instead of a doctor. The computer program compares your X-ray to 10,000 other X-rays and determines that you broke your ankle. |
| Watch monitors risk of heart attack  You bought a new watch that monitors your heart rate, breathing, physical activity, and sleep patterns. A computer program then compares your information with the medical information from thousands of other people. Based on this comparison, the watch tells you how likely you are to have a heart attack in the next two years.  Estimate cancer survival |
| Your doctor has diagnosed you with colon cancer. The cancer clinic has a computer program that uses the medical information of thousands of patients with colon cancer to estimate survival. This computer reviews your medical information and predicts you have a very low chance of surviving more than six months.  App for sadness |
| You have been feeling sad lately. A new App on your phone lets you talk about your feelings. It talks back to you with a human-like voice and gives you advice about how to feel better. The App bases its suggestions on large amounts research. It also adjusts what it tells you based on what you tell it and how you respond over time to the suggestions.  Recommend anxiety medication |
| Your doctor diagnoses you with an anxiety disorder. A computer program reviews your health records and all of the latest research about treatment options for anxiety disorders. It then suggests the best medication for you to take.  Camera device predicts pain in hospital room |
| You are staying in the hospital after a major surgery. Your room has a camera that tracks your facial expressions and movements. Based on this video, a computer program predicts when your pain is about to worsen. |

Presentation of scenarios was randomized to address the potential for order effects.
