# Supplementary material for: Exploring perceptions of healthcare technologies enabled by artificial intelligence: an online, scenario-based survey
Source: BMC Med Inform Decis Mak. 2021 Jul 20;21:221. doi: 10.1186/s12911-021-01586-8 (PMC8293482; doi:10.1186/s12911-021-01586-8)
Supplement: Supplementary file 2 — Additional file 1: Table S2. Example scenario with openness, concern, and benefit items. [file 12911_2021_1586_MOESM2_ESM.docx]

| **Table S2.**  Example Scenario with Openness, Concern, and Benefit items | | | | | | |
| --- | --- | --- | --- | --- | --- | --- |
| Scenario  You bought a new watch that monitors your heart rate, breathing, physical activity, and sleep patterns. A computer program then compares your information with the medical information from thousands of other people. Based on this comparison, the watch tells you how likely you are to have a heart attack in the next two years. | | | | | | |
| Openness item and response scale   1. How open are you to having this watch determine how likely you are to have a heart attack in the next two years? | | | | | | |
|  | 1 | 2 | 3 | 4 | 5 |  |
|  | Not at all open | Slightly open | Moderately open | Very open | Extremely open |  |
| Instructions and response scale for concern and benefit items  For each statement below, answer the following question.  Does this information make you view the watch more positively or more negatively? | | | | | | |
| 1 | 2 | 3 | 4 | 5 | 6 | 7 |
| much more negatively | more negatively | slightly more negatively | neither more positively nor negatively | slightly more positively | more positively | much more positively |
| Concern and benefit items   1. Your doctor spends much of your next appointment looking at the watch instead of talking with you. (C) 2. The watch recommends steps you can take to lower your risk of heart attack. (B) 3. A similar company was recently hacked and users’ personal information was stolen. (C) 4. A new study found that this device is not as good at predicting chances of a heart attack for minority patients. (C) 5. The company does not know exactly how the computer makes these predictions. (C) 6. In a new study, this watch was better at predicting chances of heart attack than doctors alone. (B) | | | | | | |

Underline = components of the measure. C = concern item; B = benefit item. Measure was administered via survey software so formatting and style differed from shown here.
