# Supplementary material for: Exploring perceptions of healthcare technologies enabled by artificial intelligence: an online, scenario-based survey
Source: BMC Med Inform Decis Mak. 2021 Jul 20;21:221. doi: 10.1186/s12911-021-01586-8 (PMC8293482; doi:10.1186/s12911-021-01586-8)
Supplement: Supplementary file 3 — Additional file 3. Full results of the exploratory and confirmatory factor analysis. [file 12911_2021_1586_MOESM3_ESM.docx]

**Appendix.**

**Full Results of the Exploratory and Confirmatory Factor Analysis**

The Bartlett’s test of sphericity (χ^2^ (1431) = 12494.66 p < .001) indicated it was appropriate to factor analyze the data. The Kaiser-Meyer-Olkin measure of sampling adequacy indicated the strength of the relationships among the variables was high (KMO = .93).

We used principal axis factoring with promax rotation and extracted Eigenvalues over 1. To determine how many factors to extract, we examined the scree plot and the relative variance explained by the factors. The results of the initial EFA indicated that a 3-factor solution explained 42% of the variance, with the first 2 factors explaining 19.5% and 18%, respectively. The third factor explained 4.5%. The 4 items that loaded on the third factor cross-loaded (> .30) with factor 2 and the items were conceptually meaningful with the items that loaded on factor 2. All items at this stage has factors loadings of .30 or higher on these first 3 factors. Given the considerably lower variance explained by factor 3 and meaningful cross loadings with factor 2, we conducted a second EFA extracting 2 factors to evaluate this more parsimonious model.

This 2-factor solution explained 38% of the variance. Factor 1 had 29 items with loadings of .41 or greater, one item was dropped as its loading was .30 and it cross-loaded with the second factor. Factor 2 had 24 items with loadings of .37 or greater. One item was dropped for having a cross loading below .30. Given the simple 2-factor structure, we decided to cut two scenarios to shorten the measure. We dropped two scenarios because we judged these scenarios to be most similar to the six remaining; we opted to retain the six scenarios that covered the most unique types of health and technology contexts. We also evaluated whether some items should be dropped due to low variance in responses, and dropped one additional item. In all, we dropped 16 items, and reran the EFA with the 38 remaining items. Table S3 (Additional file 4) shows the factor loadings for the EFA with the 38 items.

The final 2-factor EFA model explained 40% of the variance, with Factor 1 explaining 22% of the variance and consisting of 22 items representing concerns. Factor 2 explained 18% of the variance and consisted of 16 items representing benefits. The factors were orthogonal (uncorrelated). Cronbach’s alpha for the concern factor was .92 and alpha was .89 for the benefit factor. The key finding of our EFA was that items loaded together according to their positive or negative valence as a benefit or concern, not into the separate theoretical dimensions. Six items originally intended as reversed scored benefit or concern items loaded on the opposite factor, indicative of the concern-or-benefit response pattern.

We obtained responses from a second set of respondents (N = 467) to verify the factor structure. We performed a confirmatory factor analysis (CFA) using maximum likelihood estimation and fitting an orthogonal model. The model had acceptable fit to the observed data, χ^2^(629) = 2668.10, p < .001, CFI = .71, SRMR = .08, RMSEA = .08.
