# Supplementary material for: Exploring perceptions of healthcare technologies enabled by artificial intelligence: an online, scenario-based survey
Source: BMC Med Inform Decis Mak. 2021 Jul 20;21:221. doi: 10.1186/s12911-021-01586-8 (PMC8293482; doi:10.1186/s12911-021-01586-8)
Supplement: Supplementary file 4 — Additional file 4: Table S3. Results of the final 38-item exploratory factor analysis conducted in sample 1 (N = 469). [file 12911_2021_1586_MOESM4_ESM.docx]

| **Table S3.**  Results of the Final 38-item Exploratory Factor Analysis Conducted in Sample 1 (N = 469) | | |
| --- | --- | --- |
| **Item** | Factor 1  Concern | Factor 2  Benefit |
| 1. Your insurance company charges you an additional copay to use this program. | 0.72 |  |
| 1. Another company that makes similar watches was recently hacked and users’ personal information was stolen. | 0.70 |  |
| 1. You know there is a camera recording in your room, but you do not know how the recording will be used. | 0.68 |  |
| 1. Hackers recently stole users’ messages from a similar mental health App and posted them online. | 0.67 |  |
| 1. The company does not share exactly how the watch makes these predictions. | 0.67 |  |
| 1. If your doctor uses this computer program, the company that built it will have access to your medical record. | 0.65 |  |
| 1. Your doctor prescribes the medication the computer program recommended without discussing alternatives with you. | 0.64 |  |
| 1. There is no information provided about how your chance of survival is determined. | 0.63 |  |
| 1. Medicaid insurance does not cover the use of this computer program for its patients. | 0.63 |  |
| 1. This computer program makes more mistakes when reviewing X-rays of women compared to men. | 0.62 |  |
| 1. A new study found that using this computer program increases costs to the U.S. healthcare system. | 0.59 |  |
| 1. The camera records you in your hospital bed at all times. | 0.59 |  |
| 1. You were not told that a computer program picked the medication for you instead of your doctor. | 0.58 |  |
| 1. Your doctor spends much of your next appointment looking at the watch instead of talking with you. | 0.58 |  |
| 1. A new study found that this watch is not as good at predicting chances of a heart attack for people of color. | 0.57 |  |
| 1. A study finds that using this computer program costs the healthcare system 20% more than regular care. | 0.55 |  |
| 1. You have to pay out-of-pocket for the watch. | 0.55 |  |
| 1. The company that built this computer program will keep your medical information on their company server. | 0.54 |  |
| 1. A new study found that this program is better at picking medicines for White patients than other patients. | 0.49 |  |
| 1. A doctor told you that you broke your ankle, but not that a computer program reviewed your X-ray. | 0.48 |  |
| 1. The counseling provided by the App involves no human mental health care providers. | 0.41 |  |
| 1. You met someone else diagnosed with cancer who lived much longer than the computer program predicted. | 0.41 |  |
| 1. This App gives you advice that is easy to understand. |  | 0.71 |
| 1. The watch recommends steps you can take to lower your risk of a heart attack. |  | 0.68 |
| 1. When the program identifies signs of pain, it signals the nurse to come to your room. Your nurse sits with you and asks about your discomfort. |  | 0.68 |
| 1. Before you leave the hospital, the program provides you with information about how to manage pain without medication. |  | 0.67 |
| 1. This program allows you to get pain medications quickly when you feel pain. |  | 0.67 |
| 1. This watch is better at predicting chances of a heart attack than doctors. |  | 0.68 |
| 1. A friend told you that this App helped them to feel better after about two weeks. |  | 0.61 |
| 1. This program also emails you information about other ways to manage your anxiety. |  | 0.61 |
| 1. Using this camera and program in the hospital allows you to return to work 2 days earlier because your pain is better controlled. |  | 0.60 |
| 1. If you have a high risk of having a heart attack, the watch sends an alert to your doctor. |  | 0.60 |
| 1. Using the computer program makes your visit to urgent care shorter. |  | 0.60 |
| 1. The computer program provides a list of the pros and cons of different treatment options. |  | 0.56 |
| 1. Hospitals that use this computer program reduce their costs significantly. |  | 0.55 |
| 1. This computer program is better than doctors at identifying small breaks in a bone. |  | 0.55 |
| 1. A study showed that using this App saves the healthcare system a lot of money. |  | 0.53 |
| 1. Your medical bill will cost $100 less than if a doctor reviewed your X-ray. |  | 0.34 |
